# Supplementary material for: Self-Reconstructed Metal–Organic Framework-Based Hybrid Electrocatalysts for Efficient Oxygen Evolution
Source: Nanomaterials (Basel). 2024 Jul 9;14(14):1168. doi: 10.3390/nano14141168 (PMC11279696; doi:10.3390/nano14141168)
Supplement: Supplementary file 1 [file nanomaterials-14-01168-s001.zip › nanomaterials-3060091-supplementary.pdf]

nanomaterials

Supplementary Materials

Self-Reconstructed Metal-Organic Framework-Based Hybrid Electrocatalysts for Efficient Oxygen Evolution

Kunting Cai [1], Weibin Chen [1], Yinji Wan [2], Hsingkai Chu [1], Xiao Hai [1,]* and Ruqiang Zou,[1,]*

[1] Beijing Key Laboratory for Theory and Technology of Advanced Battery Materials, School of Materials Science and Engineering, Peking University, No. 5 Yiheyuan Road, Haidian District, Beijing 100871, China
[2] State Key Laboratory of Heavy Oil Processing, China University of Petroleum, Beijing, No.18 Fuxue Road, Changping District, Beijing 102249, China
* Correspondence: xiaohai@pku.edu.cn; rzou@pku.edu.cn

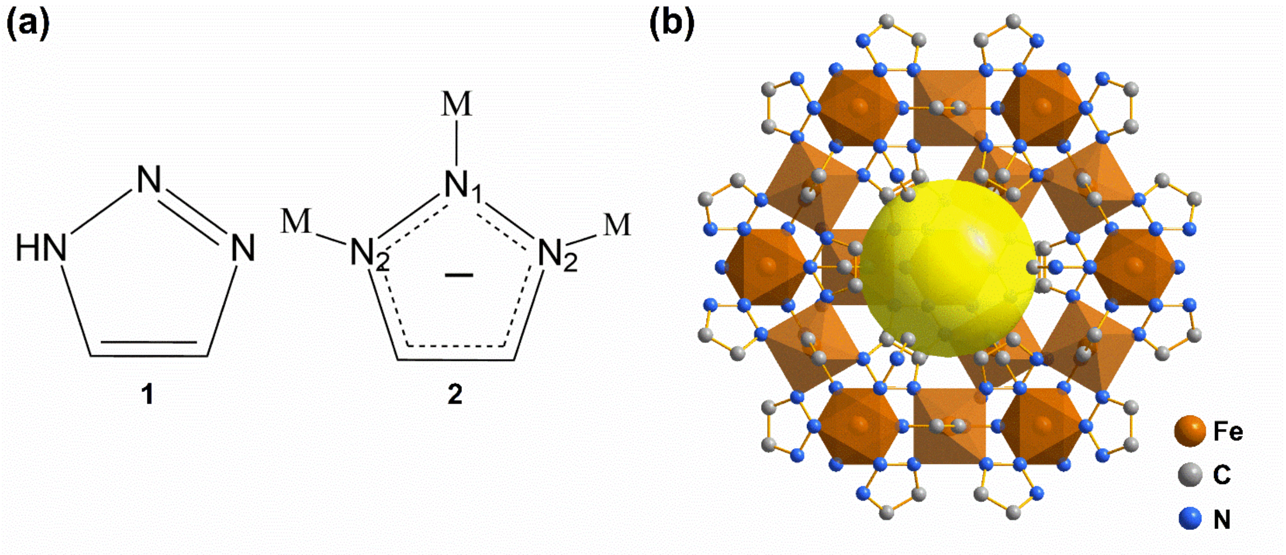

Figure S1. (a) Illustration of 1H-1,2,3-triazole molecule (**1**) deprotonates to form the triazolate anion. Every nitrogen atom (including N_1 type and N_2 type which are different from chemical environment) is able to coordinate several metal atoms in mode (**2**). (b) Illustration of MET-Fe with a pore (yellow sphere). Iron atoms are represented as brown spheres or polyhedron, nitrogen and carbon atoms are blue and grey spheres, respectively.

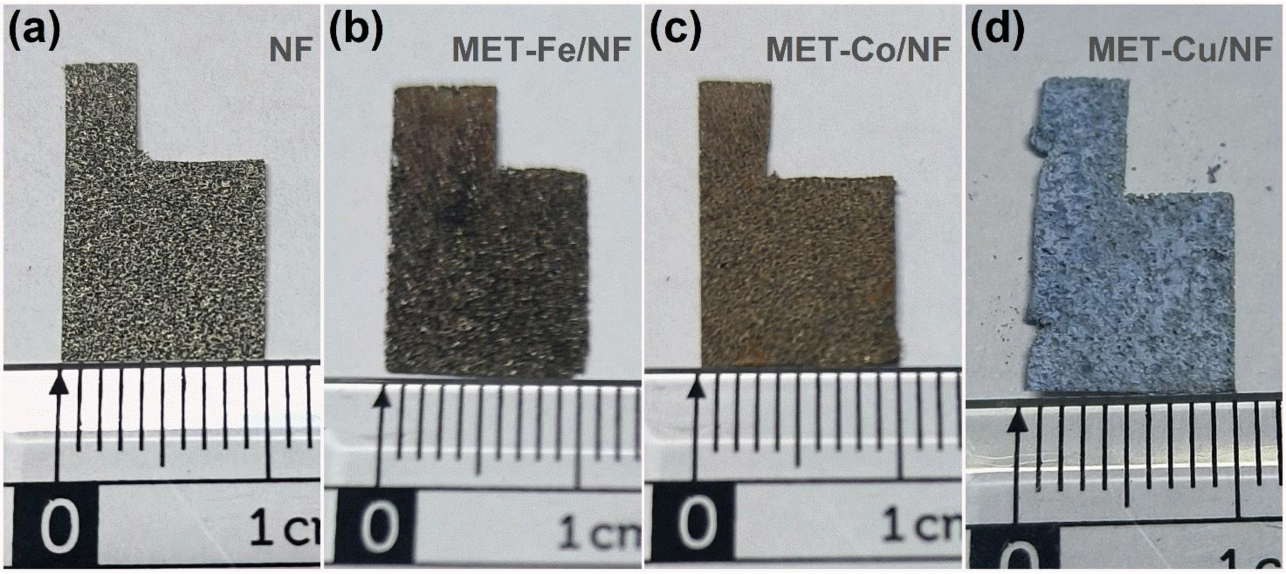

Figure S2. The photograph of (a) NF, (b) MET-Fe/NF, (c) MET-Co/NF and (d) MET-Cu/NF.

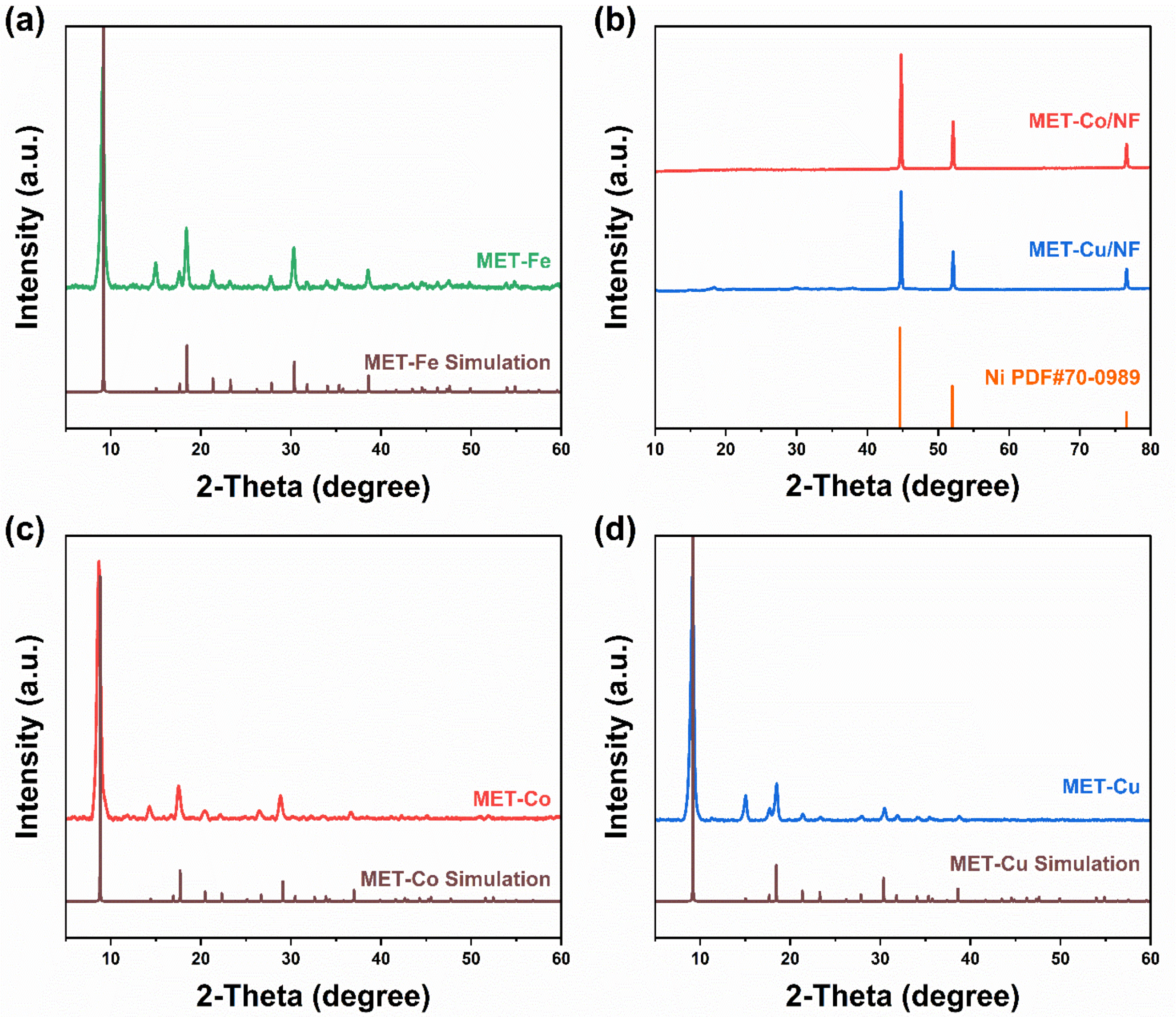

Figure S3. XRD patterns of (a) MET-Fe, (b) MET-Co/NF and MET-Cu/NF, (c) MET-Co and (d) MET-Cu.

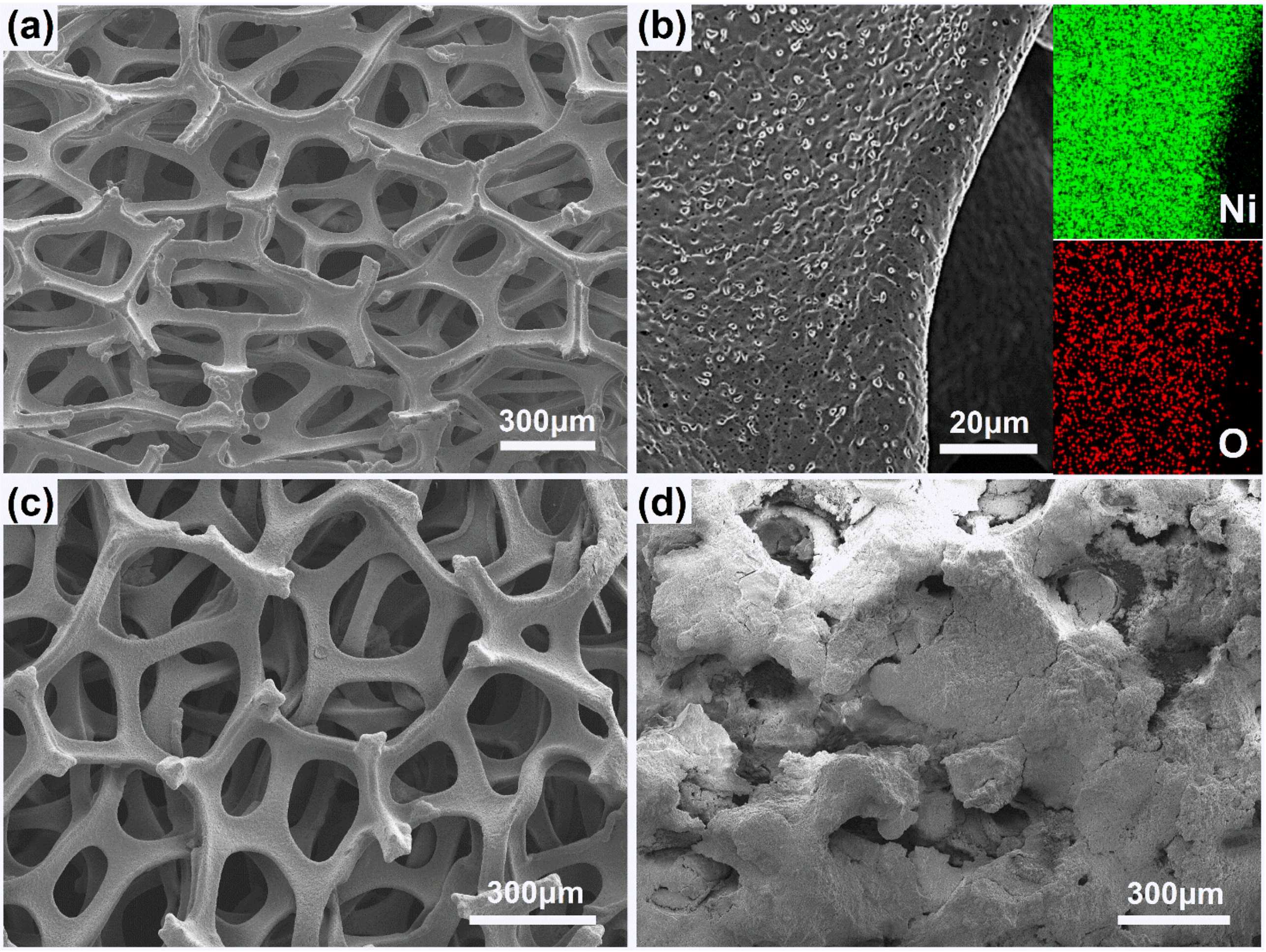

Figure S4. (a) SEM images of NF. (b) Elemental mapping images of NF. SEM images of (c) MET-Co/NF and (d) MET-Cu/NF.

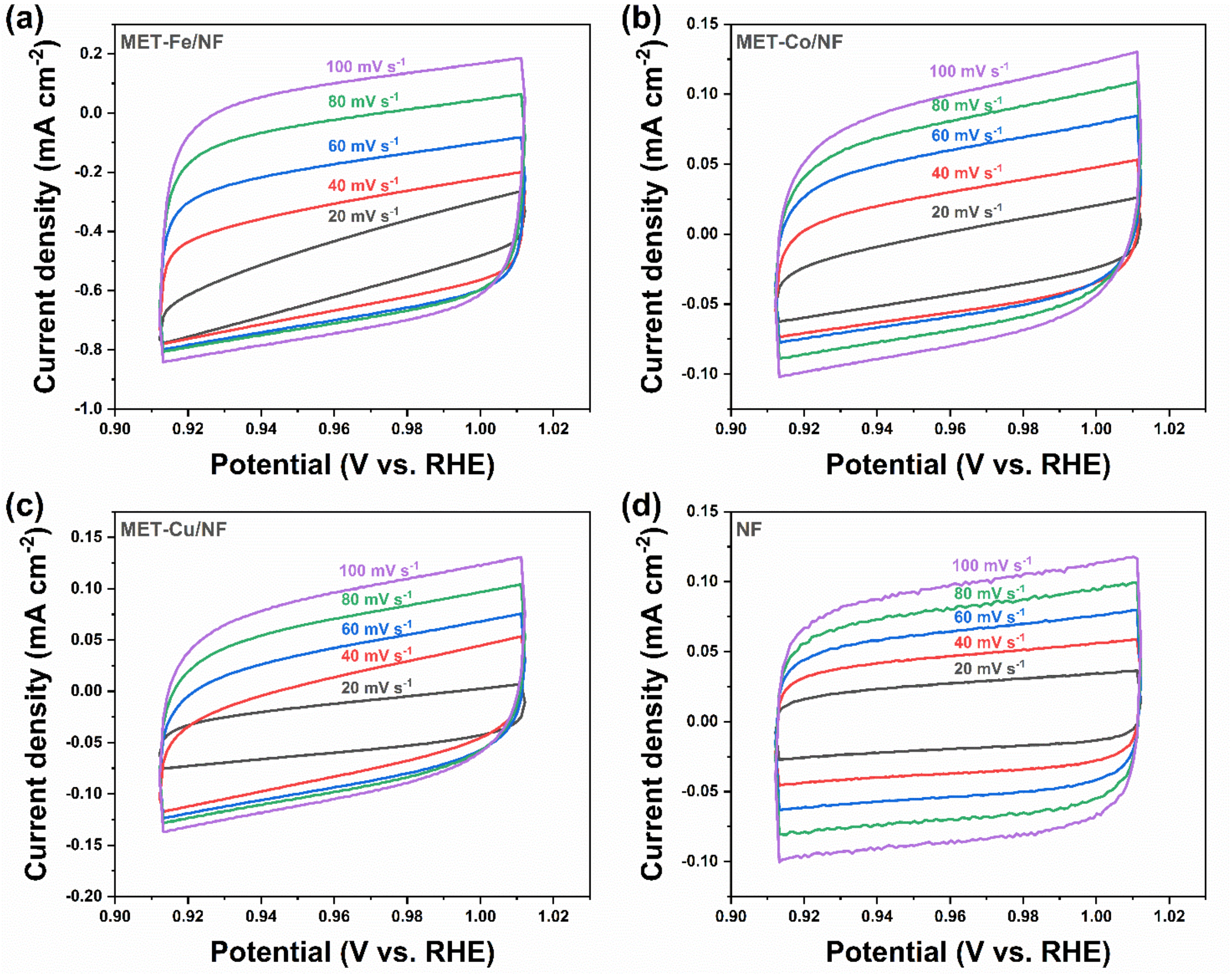

Figure S5. Cyclic voltammetry profiles at different scan rates (20-100 mV) in 1.0 M KOH for (a) MET-Fe/NF, (b) MET-Co/NF, (c) MET-Cu/NF and (d) NF.

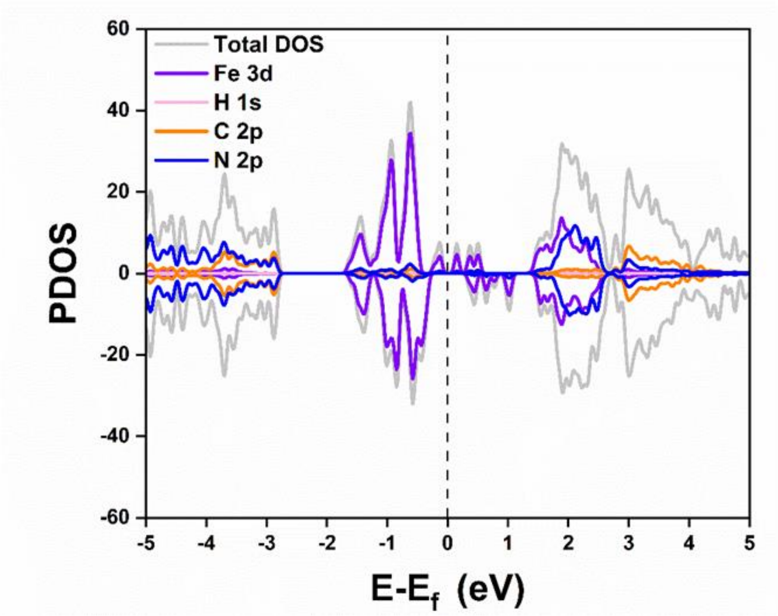

Figure S6. The projected density of states (PDOS) calculation of MET-Fe on the mainly involved elements and orbitals.

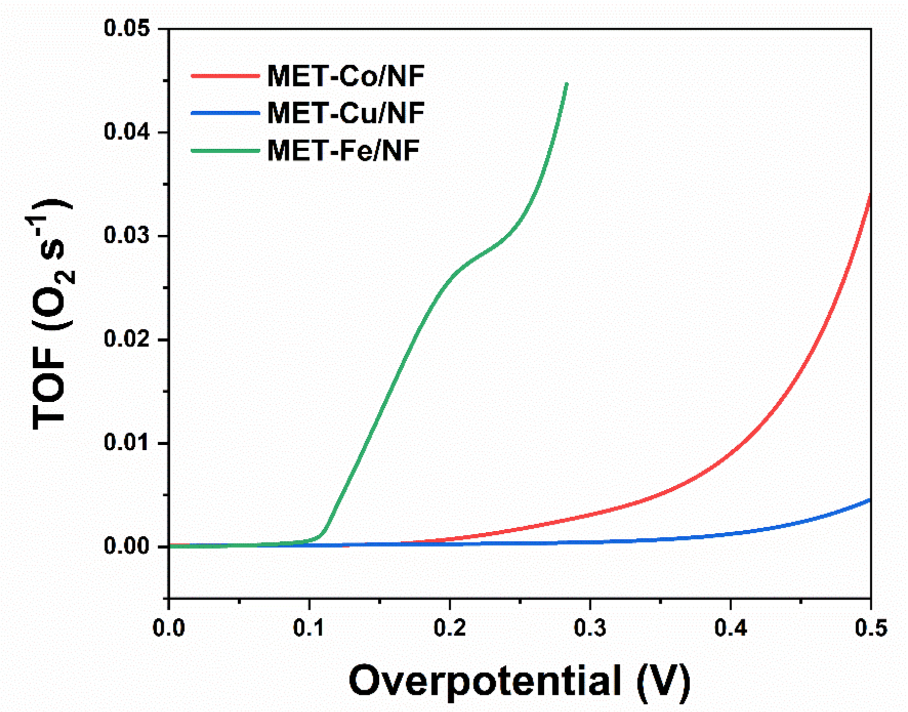

Figure S7. The turnover frequency (TOF) profiles versus overpotential of MET-Fe/NF, MET-Co/NF and MET-Cu/NF.

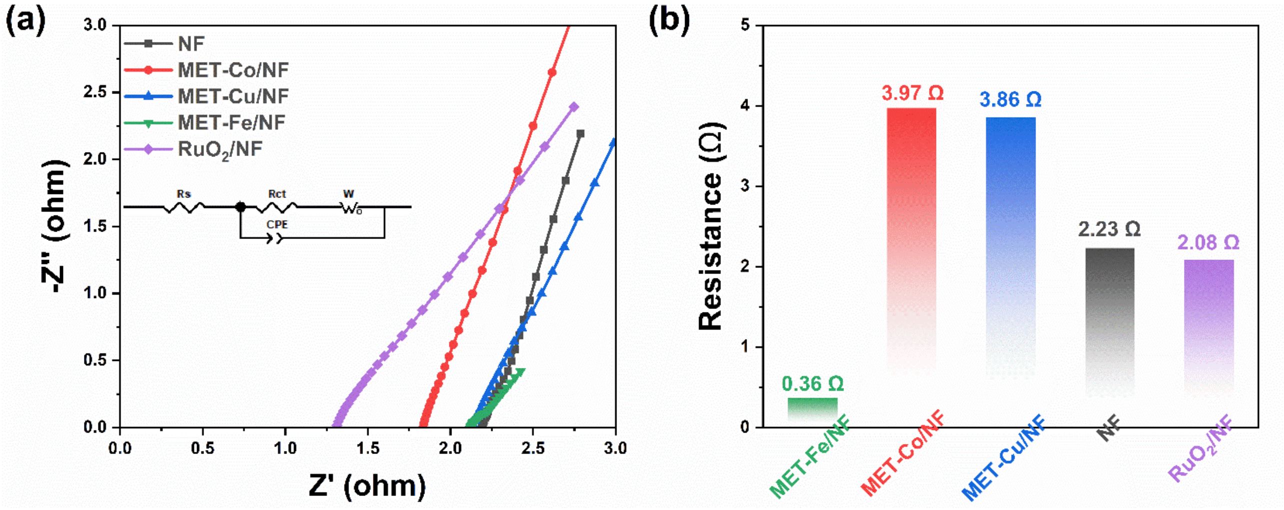

Figure S8. (a) Nyquist plots (The insert shows the equivalent circuit). (b) Charge transfer resistance based on the fitting data.

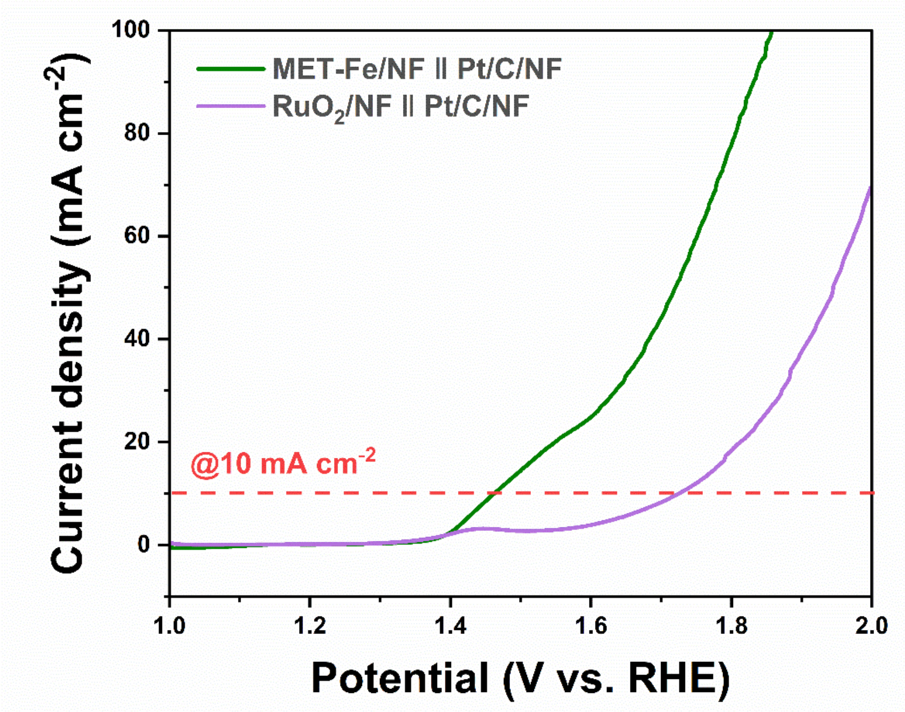

Figure S9. LSV curve comparation of overall water splitting over the MET-Fe/NF | | Pt/C/NF and RuO_2/NF | | Pt/C/NF two-electrode setups.

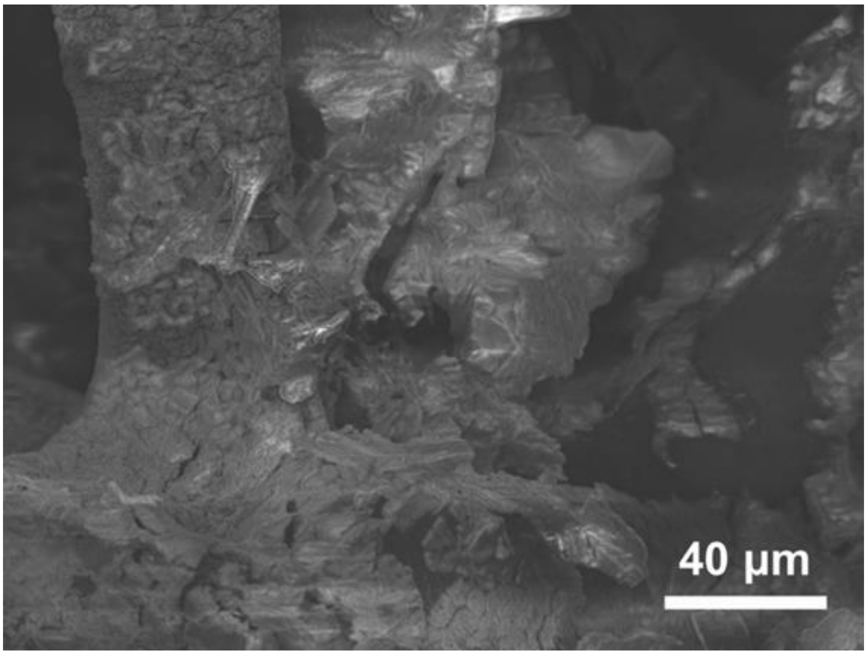

Figure S10. SEM of MET-Fe/NF after OER test.

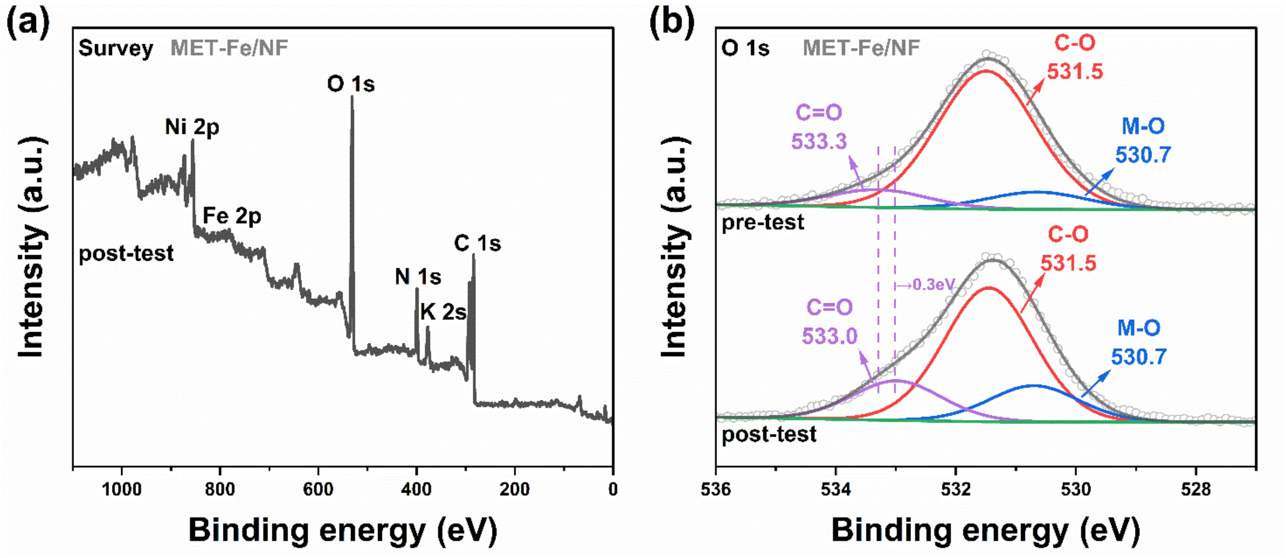

Figure S11. XPS survey spectrum of (a) MET-Fe/NF after OER test. High resolution XPS spectra of (b) O 1s of MET-Fe/NF before and after OER test.

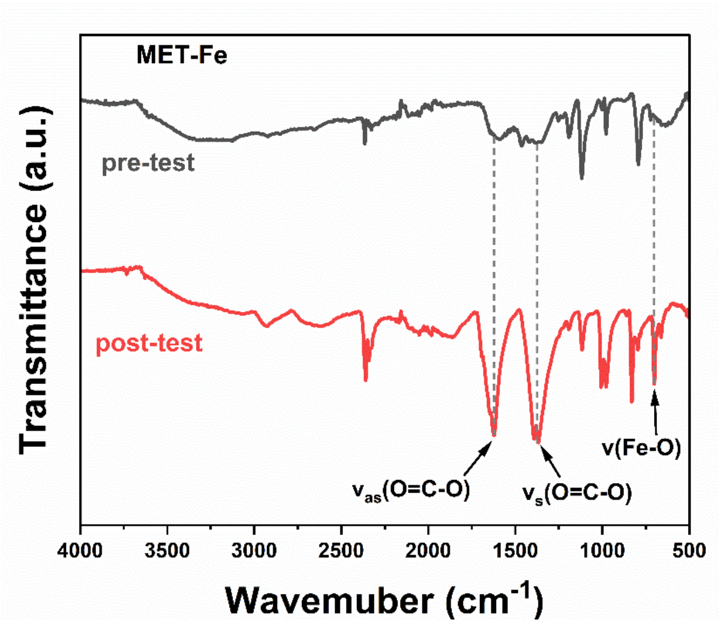

Figure S12. FT-IR spectrum of MET-Fe/NF before and after OER test.

Table S1. Comparison of OER performance with recently reported MOF-based superior electrocatalysts in 1M KOH.

Electrocatalyst	Substrate	η (mV)	Current density (mA cm^{-2})	Tafel slope (mV dec^{-1})	Ref.
**MET-Fe/NF**	**NF**	**10**	**122**	**34.5**	**This work**
FeNi DHBQ/NF	NF	10	203	40.5	[1]
Ni-NDC@NF	NF	10	249	74	[2]
NiFe-MOF-2/NF	NF	10	209	36.4	[3]
FeCoNi-btz/NF	NF	10	263	64	[4]
3A-TDC-MOF	NF	10	211	40.3	[5]
FeCoNi MOF/NF	NF	10	267	37.6	[6]
Fe-B/Fe-MOF/IF	IF	10	210	38	[7]
Cu-doped NiFe-BDC	IF	10	200	43.2	[8]
NiFe-PBA@IF	IF	10	253	47.9	[9]
S-NiFe/NFF	NNF	10	174	60	[10]
MOF-(74 + 274)@NFF	NNF	10	198	38.3	[11]

Note: NF = Nickel foam, IF = Iron foam, NNF = Nickel-iron foam.

Table S2. Comparison of over-water splitting activity performance with recently reported MOF-based superior electrocatalysts in 1M KOH.

Electrocatalyst	Current density (mA cm^{-2})	Potential (V)	Ref.
**MET-Fe/NF**||**Pt/C/NF**	**10**	**1.463**	**This work**
MOF/NF-SO || Pt/C	10	1.593	[6]
Fe–B/Fe-MOF/IF || Fe–B/Fe-MOF/IF	10	1.53	[7]
S-NiFe/NFF || S-NiFe/NFF	10	1.50	[10]
NiFeMo-MOF/NF || (M)Ni-NiOOH/NF	10	1.50	[12]
NC-2@CoO/NF || NC-2@CoO/NF	10	1.54	[13]
FcNi-BDC-H_2O_2/NF || Pt	10	1.542	[14]
$Ni_{0.67}Fe_{0.33}$-MOF/CFP || Pt/C	10	1.48	[15]

References

1. Liu, J.; Yu, Z.; Huang, J.; Yao, S.; Jiang, R.; Hou, Y.; Tang, W.; Sun, P.; Huang, H.; Wang, M. Redox-active ligands enhance oxygen evolution reaction activity: Regulating the spin state of ferric ions and accelerating electron transfer. *Journal of Colloid and Interface Science* 2023, *650*, 1182-1192, doi:https://doi.org/10.1016/j.jcis.2023.07.083.
2. Lee, M.K.; Choi, S.; Park, H.; Lee, T.H.; Lee, S.A.; Yang, J.W.; Ji, S.G.; Cheon, W.S.; Ahn, S.H.; Kim, S.Y.; et al. 2D Ni-Naphthalene-2,6-Dicarboxylic Acid Metal-Organic Framework as Electrocatalysts for Efficient Overall Water Splitting. *Energy Technology* **2023**, *11*, 2201203, doi:https://doi.org/10.1002/ente.202201203.
3. Wu, F.; Li, Q.; Guo, H.; Wang, S.; Hao, G.; Hu, Y.; Zhang, G.; Jiang, W. Modulating crystal and electronic structure of NiFe-MOFs by inorganic acid for highly efficient electrochemical water oxidation. *Dalton Transactions* **2023**, *52*, 2027-2035, doi:10.1039/D2DT02925A.
4. Li, S.; Wang, Z.; Wang, T.; Yang, Y.; Xiao, Y.; Tian, Y.; Zhu, H.; Jing, X.; Zhu, G. Preparation of Trimetallic-Organic Framework Film Electrodes via Secondary Growth for Efficient Oxygen Evolution Reaction. *Chemistry – A European Journal* **2023**, *29*, e202301129, doi:https://doi.org/10.1002/chem.202301129.
5. Wang, Q.; Ma, X.; Ma, P.; Bi, R.; Song, S. Defect-Rich, Rose-Shaped Fe2Ni1-Metal–Organic Framework Nanoarrays for Efficient Oxygen Evolution Reaction. *ACS Applied Nano Materials* **2023**, *6*, 9339-9350, doi:10.1021/acsanm.3c00962.
6. Li, W.; Jia, S.; Liu, X.; Li, Y.; Chen, T.; Yang, F.; Zhang, X. Exploration and Application of the Self-Optimization Phenomenon of a Trimetal-Based MOF Electrocatalyst in the Oxygen Evolution Reaction. *Energy & Fuels* **2023**, *37*, 8563-8572, doi:10.1021/acs.energyfuels.3c00984.
7. Zhao, S.; Deng, L.; Xiong, Y.; Hu, F.; Yin, L.; Yu, D.; Li, L.; Peng, S. Engineering metal-organic framework nanosheets with electronically modulated in-plane heterojunctions for robust high-current-density water splitting. *Science China Materials* **2023**, *66*, 1373-1382, doi:10.1007/s40843-022-2274-7.

8. Hu, F.; Yu, D.; Zeng, W.-J.; Lin, Z.-Y.; Han, S.; Sun, Y.; Wang, H.; Ren, J.; Hung, S.-F.; Li, L.; et al. Active Site Tailoring of Metal-Organic Frameworks for Highly Efficient Oxygen Evolution. *Advanced Energy Materials* **2023**, *13*, 2301224, doi:https://doi.org/10.1002/aenm.202301224.
9. Wei, L.; Meng, D.; Mao, J.; Jiang, Q.; Huang, H.; Tang, J. Assembly of NiFe-PBA nanoparticles on nanoflower-like NiFe-PBA@IF as enhanced oxygen evolution electrocatalyst at room temperature. *Molecular Catalysis* **2023**, *544*, 113126, doi:https://doi.org/10.1016/j.mcat.2023.113126.
10. Wu, L.; Feng, J.; Zou, Z.; Song, K.; Zeng, C. Formation of feathery-shaped dual-function S-doped FeNi-MOFs to achieve advanced electrocatalytic activity for OER and HER. *Journal of Electroanalytical Chemistry* **2023**, *935*, 117365, doi:https://doi.org/10.1016/j.jelechem.2023.117365.
11. Jiang, Y.; Chen, T.-Y.; Chen, J.-L.; Liu, Y.; Yuan, X.; Yan, J.; Sun, Q.; Xu, Z.; Zhang, D.; Wang, X.; et al. Heterostructured Bimetallic MOF-on-MOF Architectures for Efficient Oxygen Evolution Reaction. *Advanced Materials* **2023**, *n/a*, 2306910, doi:https://doi.org/10.1002/adma.202306910.
12. Yin, Z.; Liang, J.; Zhang, Z.; Luo, H.; Zhou, J. Construction of superhydrophilic metal-organic frameworks with hierarchical microstructure for efficient overall water splitting. *Journal of Colloid and Interface Science* **2022**, *623*, 405-416, doi:https://doi.org/10.1016/j.jcis.2022.05.057.
13. Van Phuc, T.; Jana, J.; Ravi, N.; Kang, S.G.; Chung, J.S.; Choi, W.M.; Hur, S.H. Highly active Ni/Co-metal organic framework bifunctional electrocatalyst for water splitting reaction. *International Journal of Hydrogen Energy* **2022**, *47*, 22787-22795, doi:https://doi.org/10.1016/j.ijhydene.2022.05.097.
14. Liu, H.; Zhang, T.; Cui, D.; Zheng, Y.; Cheng, Y.; Wang, G.; Chen, L. Defective ferrocene-based metal–organic frameworks for efficient solar-powered water oxidation via the ligand competition and etching effect. *Journal of Colloid and Interface Science* **2024**, *657*, 664-671, doi:https://doi.org/10.1016/j.jcis.2023.12.024.
15. Zhang, S.; Huang, Z.; Isimjan, T.T.; Cai, D.; Yang, X. Accurately substituting Fe for Ni2 atom in Ni-MOF with defect-rich for efficient oxygen evolution reaction: Electronic reconfiguration and mechanistic study. *Applied Catalysis B: Environmental* **2024**, *343*, 123448, doi:https://doi.org/10.1016/j.apcatb.2023.123448.

Disclaimer/Publisher's Note: The statements, opinions and data contained in all publications are solely those of the individual author(s) and contributor(s) and not of MDPI and/or the editor(s). MDPI and/or the editor(s) disclaim responsibility for any injury to people or property resulting from any ideas, methods, instructions or products referred to in the content.
